# Think Digital—The New Era in the Dentistry Field

**DOI:** 10.3390/jcm11144073

**Published:** 2022-07-14

**Authors:** Adolfo Di Fiore

**Affiliations:** Department of Neurosciences, Section of Prosthodontic and Digital Dentistry, School of Dentistry, University of Padova, 35122 Padova, Italy; adolfo.difiore@unipd.it

In recent years the dental field has evolved incredibly due to the introduction of digital technology. Thanks to new devices such as intraoral and facial scanners, different clinical procedures have been facilitated by reducing clinical steps and operative time [1,2]. The most changes have emerged in the discipline of prosthetic dentistry, where advances in technology have provided the ability to realize a crown or an inlay in a few hours. However, such simplification is not of interest only to the daily clinic approach, but also to diagnosis, clinical training, and manufacturing methods. The three-dimensional environment allows clinicians and students to analyze bone quality and quantity, assess the tooth preparation, and to design a new smile. Moreover, the choice to realize types of dental prostheses with different techniques has opened a new scenario in dental materials science. New dental materials have been developed, improving the esthetics, and providing a better potential for long-term survival and stability. Although the development of material through subtractive manufacturing presents better mechanical and surface characteristics than additive manufacturing [3], the future will be additive. This manufacturing method is an eco-friendly technology, due to the lower environmental impact, the reduced waste of materials, and the use of recyclable materials. However, the question of whether digital dentistry is the past, present, or future remains without an answer. A unanimous consensus is not present among clinicians. Some prefer the use of a conventional workflow or use a combined workflow, whereas others apply a completely digital workflow. The reasons are several, but I think that this difference is mainly attributable to the reluctance of clinicians to change their daily workflow. Where the conventional workflow results in the omission of some preparation errors, the digital workflow does not. All clinical steps must be executed with the utmost accuracy. For example, the incorrect management of an interim crown does not allow the control of many problems associated with digital impressions, such as localized bleeding, the retraction technique, and the limits of scanners to acquire the subgingival vertical finish line. Indeed, only high-quality dentistry can take advantage of digital dentistry, more so than poor-quality dentistry. It is fundamental to remember that the patient is the most important person in dental treatment, and therefore, must be the first person that benefits from digital dental procedures. For all clinicians and students, I give one suggestion: think digital for better daily dentistry.

## References

[1] Di Fiore A., Vigolo P., Graiff L., Stellini E. (2018). Digital vs Conventional Workflow for Screw-Retained Single-Implant Crowns: A Comparison of Key Considerations. Int. J. Prosthodont..

[2] Di Fiore A., Meneghello R., Graiff L., Savio G., Vigolo P., Monaco C., Stellini E. (2019). Full arch digital scanning systems performances for implant-supported fixed dental prostheses: A comparative study of 8 intraoral scanners. J. Prosthodont. Res..

[3] Di Fiore A., Meneghello R., Brun P., Rosso S., Gattazzo A., Stellini E., Yilmaz B. (2021). Comparison of the flexural and surface properties of milled, 3D-printed, and heat polymerized PMMA resins for denture bases: An in vitro study. J. Prosthodont. Res..

